# Human Papillomavirus 16 Infection and *TP53* Mutation: Two Distinct Pathogeneses for Oropharyngeal Squamous Cell Carcinoma in an Eastern Chinese Population

**DOI:** 10.1371/journal.pone.0164491

**Published:** 2016-10-17

**Authors:** Zhen Wang, Rong-Hui Xia, Dong-Xia Ye, Jiang Li

**Affiliations:** 1 Department of Oral Pathology, ninth People's Hospital, Shanghai Jiao Tong University, School of Medicine, Shanghai Key Laboratory of Stomatology, Shanghai, P. R. China; 2 Department of Oral Maxillofacial Surgery, ninth People's Hospital, Shanghai Jiao Tong University, School of Medicine, Shanghai Key Laboratory of Stomatology, Shanghai, P. R. China; Georgetown University, UNITED STATES

## Abstract

**Objectives:**

To investigate the clinicopathological characteristics, human papillomavirus (HPV) infection, p53 expression, and *TP53* mutations in oropharyngeal squamous cell carcinoma (OPSCC) and determine their utility as prognostic predictors in a primarily eastern Chinese population.

**Methods:**

The HPV infection status was tested via p16INK4A immunohistochemistry and validated using PCR, reverse blot hybridization and *in situ* hybridization (ISH) in 188 OPSCC samples. p53 expression levels and *TP53* gene mutations were assessed through immunohistochemistry and sequencing, respectively. Clinicopathological characteristics and follow-up information were collected. Overall survival was estimated using the Log-rank test.

**Results:**

Overall, 22 of the 188 OPSCC samples were associated with HPV infection. HPV16 was identified in all 22 samples, whereas no samples were positive for HPV18. All 22 HPV-associated OPSCC samples were p53 negative and lacked *TP53* mutations. HPV16 positivity, female patients, non-smokers, and patients with histological grade I and stage N0 diseases showed better overall survival (*p =* 0.009, 0.003, 0.048, 0.009, and 0.004, respectively). No significant differences in overall survival between smoking and non-smoking patients were observed in the HPV-associated OPSCC group. Patients without mutations in *TP53* exons 5–8 had better prognoses (*p =* 0.031) among the 43 sequenced specimens. Multivariate analysis indicated that HPV16 infection status (*p =* 0.011), histological grade (*p =* 0.017), and N stage (*p =* 0.019) were independent prognostic factors for patients with OPSCC.

**Conclusions:**

Distinct from the situation in Europe and America, for the patients with OPSCC in this study, HPV16 infection was relatively low, although it was still the most important independent prognostic predictor for the disease. In addition to the high smoking and drinking rate in this population, HPV16 infection and *TP53* dysfunction appear to be two distinct pathogens for OPSCC patients in the eastern Chinese population.

## Introduction

Recently, human papillomavirus (HPV)–associated oropharyngeal squamous cell carcinoma (OPSCC) has been recognized as a unique subset of head and neck squamous cell carcinomas (HNSCC). HPV infection has been implicated as a causative factor in OPSCC by the World Health Organization [1], with an infection rate varying from 36.5% to 90% in some studies [2–5], whereas tobacco and alcohol consumption are important pathogenic factors in non HPV-associated OPSCC [6–8].

Based on the prevalence of OPSCC in European and American populations, it was estimated that 72% to 96.1% of HPV-associated OPSCC patients present with high-risk type HPV16 infections [9,10]. Other HPV subtypes, such as HPV18, 31, 33, 35, 45, 51, 52, 56, 58, 59, and 68, are rare, although they can also be detected in some samples [2]. It was reported that HPV-associated OPSCC patients were more sensitive to radiotherapy and chemotherapy and exhibited lower recurrence rates, longer overall survival (OS) times, and better prognoses than HPV-negative patients [10,11]. The percentage of patients who smoked or drank was significantly higher among the HPV-negative HNSCC group, and these patients showed worse prognoses than HPV-associated HNSCC patients. HPV-negative HNSCC patients also showed higher tumor invasiveness and drug resistance, which were closely associated with one or more gene mutations [12]. *TP53* mutations were among the most common alterations found in HPV-negative HNSCC samples [13]. Indeed, the low *TP53* mutation rate and reversibility of *TP53* dysfunction could be the reason that HPV-associated HNSCC patients show better radio- and chemo-sensitivity and preferable prognoses [14,15].

Associations between the epidemiology of HPV infection and OPSCC tumorigenesis have been observed worldwide, although they have mainly been studied in developed countries [16]. Compared with Western countries, there has been much less research on this newly recognized disease in Asian nations. There have been some relevant reports from Asian regions such as Japan, South Korea, and Taiwan [17–21]. However, only one scientific paper, relying on just 66 samples, has been published in English on the clinicopathological characteristics of OPSCC in Mainland China [22]. Most recently, there was a report investigating HPV-associated OPSCC in Hong Kong, China [23]. However, the HPV infection status, clinicopathological characteristics, and *TP53* mutation rate in OPSCC patients remain unclear in other regions of China. Therefore, using a relatively large sample size, we chose to determine the HPV infection status and *TP53* mutation rate, as well as analyze the association between clinicopathological characteristics and outcome, in OPSCC patients in an eastern Chinese population.

## Materials and Methods

### Ethical Statement

Permission was obtained from the Independent Ethics Committee of Shanghai 9th People’s Hospital affiliated with Shanghai JiaoTong University, School of Medicine. All specimens were collected after signed, informed consent was obtained from all study participants. The ethical review board approved the consent procedure & execution of this project.

### Tumor Sample Collection and Patient Information

**Fig 1:**
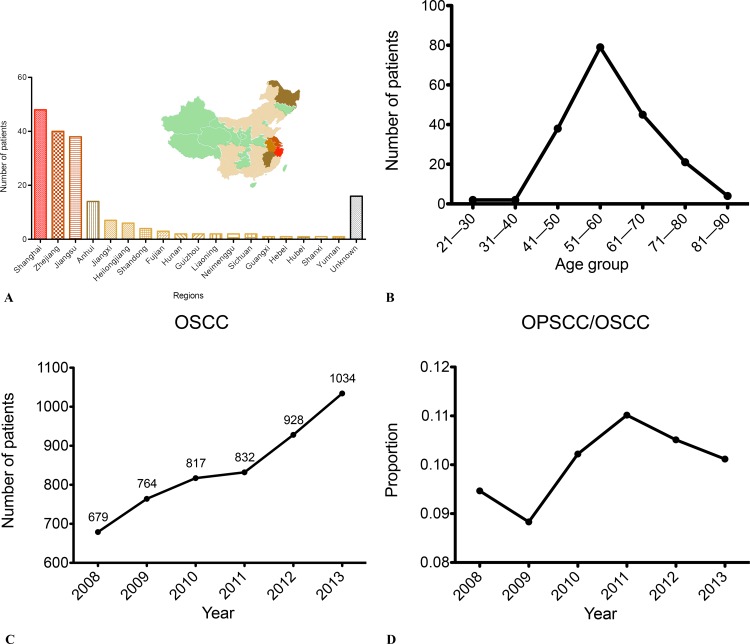
The Incidence of OPSCC from 2008 to 2013. (A) The distribution of OPSCC patients treated at Shanghai 9th People’s Hospital in Mainland China. (B) The age distribution of the 188 primary OPSCC patients. (C) The number of cases of primary OSCC patients each year from 2008 to 2013. (D) The proportional trend of OPSCC numbers relative to OSCC each year from 2008 to 2013.

A total of 188 primary OPSCC patients diagnosed at the Department of Oral Pathology in Shanghai 9th People’s Hospital affiliated with Shanghai JiaoTong University School of Medicine between January 2008 and April 2014 were enrolled in the current study. Based on the International Classification of Diseases version 9 (ICD-9), all malignant tumors that originated from oropharyngeal subsites were included, encompassing tumors of the oropharynx (ICD-9-146), base of the tongue (ICD-9-141.1), tonsils (ICD-9-141.6), and soft palate (ICD-9-145.3). The anatomical codes were verified using all available clinical and imaging records. Samples from 188 patients were collected from archived, formalin-fixed, and paraffin-embedded (FFPE) OPSCC specimens from the Department of Oral Pathology in Shanghai 9th People’s Hospital affiliated with Shanghai JiaoTong University School of Medicine between January 2008 and April 2014, and the experimental research was conducted between April 2013 and March 2016. Forty-three of the 188 FFPE samples had normal controls from tumor-free gland tissue and were used for further sequencing of the *TP53* exons 5–8. In total, 88.30% (151/171) of all patients were from eastern China, and the other 9.94% (17/171) patients were from other regions of Mainland China or had an unknown origin (Fig 1A). Determination of the primary location of the tumors was performed using a combination of clinical and pathological diagnoses. Clinicopathological data, including age, gender, region, tumor location, histological grade, TNM stage, and smoking and drinking history, were obtained from medical charts and pathological reports. All clinical and imaging records for the patients were well preserved in the respective departments and were available for access during and after data collection.

### Radiochemotherapy Status

A total of 187 patients (99.47%, 187/188) primarily received surgical treatment combined with adjuvant radiotherapy and/or chemotherapy; of these, 100 cases (53.19%, 100/188) received radiotherapy treatment, and 39 cases (20.74%, 39/188) received chemotherapy treatment. A total of 5 cases (2.66%, 5/188) received postoperative radiotherapy, 18 cases (9.57%, 18/188) received preoperative induction chemotherapy, and 9 cases (4.79%, 9/188) received additional postoperative radiochemotherapy.

### DNA Sample Extraction

According to the sizes of the different tissues, 5 to 10 pieces of 10 μm-thick paraffin sections were obtained from each tumor sample. If the polymerase chain reaction (PCR) was positive in two contiguous samples, a single blank paraffin section was inserted into the contiguous positive sample pieces, and the PCR detection was performed again in order to prevent false positives due to cross contamination. DNA samples from the 188 OPSCC paraffin section specimens were extracted using the QIAamp DNA FFPE Tissue Kit (Qiagen, Hilden, Germany) according to the manufacturer’s instructions and then stored at -20°C. All reagents were newly opened, and all consumable supplies were disposable nuclease-treated products.

### PCR Detection

The PCR positive controls were two DNA samples extracted from known HPV16- and HPV18-positive cervical cancer specimens. The negative control was a DNA sample from normal muscle tissue. The blank control was deionized water. The PCR reaction reagent was purchased from Takara Biotechnology (Takara Biotechnology, Dalian, China). The primer sequences and reaction conditions were provided in S1 Table.

### HPV Genotyping

HPV genotyping was conducted by using Human Papillomavirus Genotyping Kit (Yaneng Bio Technology, Shenzhen, China) which can identify 23 HPV subtypes, including 18 high-risk types (HPV 16, 18, 31, 33, 35, 39, 45, 51, 52, 53, 56, 58, 59, 66, 68, 73, 82 and 83) and 5 low-risk types (HPV 6, 11, 42, 43 and 44).

### IHC and Scoring

4-micron tissue sections were completely deparaffinized and hydrated. Antigen retrieval was performed in 0.01 M citric acid buffer (pH 6.0) in a hot water bath for 20 min. Endogenous peroxidases were inactivated by incubating the tissues in 3% H_2_O_2_ at room temperature for 20 min. The sections were then incubated with a primary p16INK4A monoclonal antibody (BD Biosciences, Pharmingen, CA, USA; 1:150) and a p53 monoclonal antibody (DO-7, Dako Agilent Technologies, DK; 1:150) at 4°C overnight. Signals were detected using the Envision system (Dako, Carpinteria, CA, USA) after incubation with secondary antibodies at room temperature for 30 min. HPV-associated cervical cancer sections were used as the positive control in the p16INK4A IHC assay, and known positive p53-expressing breast cancer sections were used as the positive control in the p53 IHC assay; normal mucosa specimens were used as the negative control. The primary antibody was replaced with PBS in the blank control.

The p16INK4A protein is expressed in the cytoplasm and nucleus. The p16INK4A immunohistochemistry results were considered "positive" when greater than 70% of tumor cells were positively stained [24]. The p53 protein is expressed in the nucleus, and results were considered "positive" when greater than 30% of tumor cells were positively stained [25].

### *In Situ* Hybridization (ISH)

Tissue sections (4-μm thick) were prepared. The tissues were completely deparaffinized using xylenes and rehydrated in distilled water. For antigen and nucleic acids retrieval, the tissues were heated in a hybridization enhancement solution using a microwave oven for 30 min. The HPV16/18 E6/E7 nucleic hybridization probe (Triplex international Bioscience, Fujian, China; 20 μl) was dripped onto the tissues. After denaturation at 95°C for 5 min, the tissues were incubated at 37°C for 16 h. Signals were developed in the tissues using DAB from the *in situ* hybridization developing reagent kit (Triplex international Bioscience, Fujian, China). A cervical cancer specimen with known HPV infection was used as the positive control. A muscle tissue sample from a patient with a non-HPV-associated disease was used as the negative control. The presence of yellow-brown granules in the nucleus was considered indicative of positive hybridization.

### Sanger Sequencing of *TP53* Exons 5–8

*TP53* exons 5–8 specific primers were used for the amplification of the sample DNA from the target areas. The target fragments were separated and purified using agarose electrophoresis for Sanger sequencing and statistical analysis.

### Statistical Analysis of Data

Statistical analyses were performed using SPSS 18.0 (SPSS, Chicago, IL). The results were compared using the χ^2^ test or Fisher’s exact test. Continuous variables were compared using the t test or Wilcoxon rank-sum test. Survival curves were depicted using the Kaplan-Meier method and compared using the log-rank test. Cox regression analysis was used for multivariate analyses. For all statistical tests, p values of ≤0.05 were considered significant.

## Results

### Patients and Clinicopathological Characteristics

**Fig 2:**
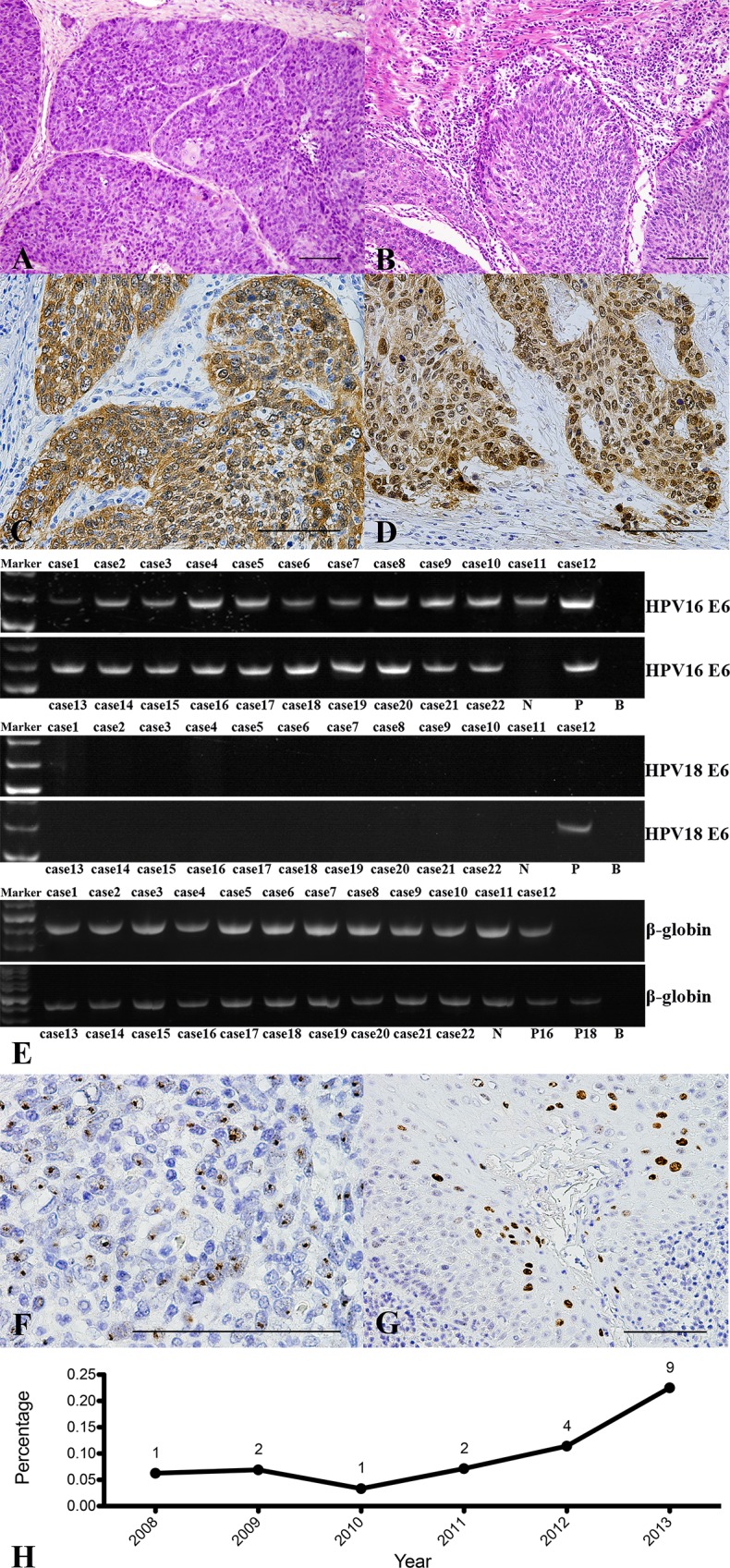
Histologic Features of HPV-Associated OPSCC and HPV Status. (A, B) HE staining of HPV-associated OPSCC with poorly differentiated tumor epithelial nests and a large number of lymphocytic infiltrations (200×). (C, D) Strong diffuse brown signals showing p16INK4A expression in the tumor cell plasma and nuclei by immunohistochemistry in ≥70% of malignant cells. (E) Native PAGE results for HPV16/18 E6: N, negative; P, positive; B, Blank; P16, HPV16 positive control; P18, HPV18 positive control. (F) 1000× magnification (hybridization dots within tumor cell nuclei, inset), integrated form. (G) 400× magnification (diffuse hybridization signals within tumor cell nuclei, inset), episomal form. (H) The proportion of HPV16-associated OPSCC/OPSCC cases from 2008 to 2013.

Of the 188 patients, 89.36% (168/188) were men, and 10.64% (20/188) were women, ranging in age from 28 to 91 years, with a mean age of 58 years. The peak age range at disease onset was 51–60 years, which accounted for 42.02% (79/188) of the total cases (Fig 1B). Between 2008 and 2014, the number of patients diagnosed with OPSCC amounted to 3.85% of all oral squamous cell carcinoma (OSCC) cases, with OSCC and OPSCC cases showing an increasing trend (Fig 1C). The proportion of OPSCC/OSCC cases ranged from 8.83% to 10.51% over the past six years, without any significant differences year to year (Fig 1D). The base of the tongue was the most common tumor site, accounting for 39.89% (75/188) of all OPSCC cases (S2 Table). Of all the patients, 10.11% (19/188) had histological grade I disease, 69.68% (131/188) had grade II disease, and 20.21% (38/188) had grade III disease. Histological subtype analysis revealed that some patients had unusual histologic expression, which primarily manifested as poorly differentiated tumor epithelial nests with a large number of lymphocytic infiltrations (Fig 2A and 2B). In 45.21% (85/188) cases, we observed lymph node metastasis in the neck. Information about smoking and drinking habits were missing for 10 of the patients, so based on data from 178 OPSCC patients, 65.17% (116/178) had a history of smoking, and 50.00% (89/178) had a history of drinking (Table 1).

**Table 1:** The Distribution of Different Clinicopathological Features from the 188 Primary OPSCC Cases Between 2008 and 2014.

	Time period							Overall	Statistical significance (2008–2013)
	2008–2009	2009–2010	2010–2011	2011–2012	2012–2013	2013–2014	2014.1–4		
**Patient/tumor data**									
No. of patients	15	29	29	27	35	40	13	188	
HPV Negative	14	27	28	25	31	30	11	166	NS
HPV Positive	1	2	1	2	4	10	2	22	
**Age at diagnosis, y**									
Mean	57	58	60.	58	57	59	59	58	NS
Median	57	55	59	57	56	58	58	58	
**Sex**									
Male	13	27	24	24	31	39	10	168	NS
Female	2	2	5	3	4	1	3	20	
**Tumor site**									
Base of tongue	7	8	12	14	14	12	7	74	NS
Oropharynx (not further specified)	3	11	8	5	9	14	4	54	
Soft palate	5	9	8	6	11	10	1	50	
Tonsil	0	1	1	2	1	4	1	10	
^**a**^**Smoking**									
Smoker	10	22	14	15	21	26	8	116	NS
Nonsmoker	4	7	13	6	14	13	5	62	
Unknown	1	0	2	6	0	1	0	10	
^**b**^**Alcohol consumption**									
Drinker	6	14	11	14	20	17	7	89	NS
Nondrinker	8	15	16	7	15	22	6	89	
Unknown	1	0	2	6	0	1	0	10	
**Pathological grades**									
1	2	4	4	2	5	1	1	19	NS
2	11	18	18	20	26	28	10	131	
3	2	7	7	5	4	11	2	38	
**Nodal stage**									
Negative	6	17	17	12	17	27	7	103	NS
Positive	9	12	12	15	18	13	6	85	
**Clinical stage**									
I~II	4	16	16	12	16	26	6	96	NS
III~IV	11	13	13	15	19	14	7	92	

NS, Not Significant

^a^Former/current smokers were defined as at least one pack-year history of smoking.

^b^Positive alcohol use was defined as current alcohol use of more than one drink per day for one year (12 ounces of beer with 5% alcohol, five ounces of wine with 12–15% alcohol, or one ounce of liquor with 45–60% alcohol). All other patients were classified as negative alcohol users.

### Correlations between HPV Status and Clinicopathological Parameters

Immunohistochemical results showed that 21.28% (40/188) cases were positive for p16INK4A (Fig 2C and 2D). PCR analysis to detect HPV16 and HPV18 DNA showed that 11.70% (22/188) cases were positive for HPV16, whereas no cases were positive for HPV18 (Fig 2E). HPV genotyping to detect 23 HPV genotypes showed no other HPV subtypes were observed, except for HPV16. The HPV genotyping result was consistent with the PCR method and only 22 HPV16 positive cases were detected. HPV16 and 18 infections were verified by *in situ* hybridization, and the results were fully consistent with the PCR results (Fig 2F and 2G). p16INK4A positivity was strongly correlated with HPV16 infection in this cohort (*p<*0.001). Statistical analysis of the number of HPV-positive patients from each year between 2008 and 2013 showed that the percentage of primary OPSCC patients with HPV infection was stable at less than 10% per year. Beginning in 2012, the HPV infection rate increased until it reached 22.5% in 2013 (Fig 2H). Significantly higher HPV infection rates were detected in male patients (*p =* 0.007), patients with tonsil lesions (*p<*0.001), non-drinkers (*p =* 0.037), and patients with histological grade III (*p =* 0.017) and p16INK4A-positive samples (*p<*0.001). HPV infection and other clinicopathological characteristics such as age, smoking, and clinical stage were not significantly correlated (Table 2).

**Table 2:** The Distribution of Different Clinicopathological Features from HPV16-Positive Cases Among 188 Primary OPSCC Patients.

	HPV Status		Overall Total	Statistical significance
	HPV negative	HPV positive		
**Patient/tumor data**				
No. of patients	166	22	188	
**Age at diagnosis, y**				
Mean	58.85	54.86	58.5	NS
Median	58	55.5	58	
**Sex**				
Male	152	16	168	0.007
Female	14	6	20	
**Tumor site**				
Base of tongue	68	6	74	<0.001
Oropharynx (not further specified)	48	6	54	
Soft palate	47	3	50	
Tonsil	3	7	10	
**Smoking**				
Smoker	106	10	116	NS
Nonsmoker	51	11	62	
Unknown	9	1	10	
**Alcohol consumption**				
Drinker	83	6	89	0.037
Nondrinker	74	15	89	
Unknown	9	1	10	
**Pathological grades**				
1	19	0	19	0.017
2	118	13	131	
3	29	9	38	
**Nodal stage**				
Negative	95	8	103	NS
Positive	71	14	85	
**Clinical stage**				
I~II	88	8	96	NS
III~IV	78	14	92	
**P16INK4A**				
Negative	148	0	148	<0.001
Positive	18	22	40	

NS, Not Significant

### Correlations between p53 IHC Staining, Mutations in *TP53* Exons 5–8, and Clinicopathological Parameters

**Fig 3:**
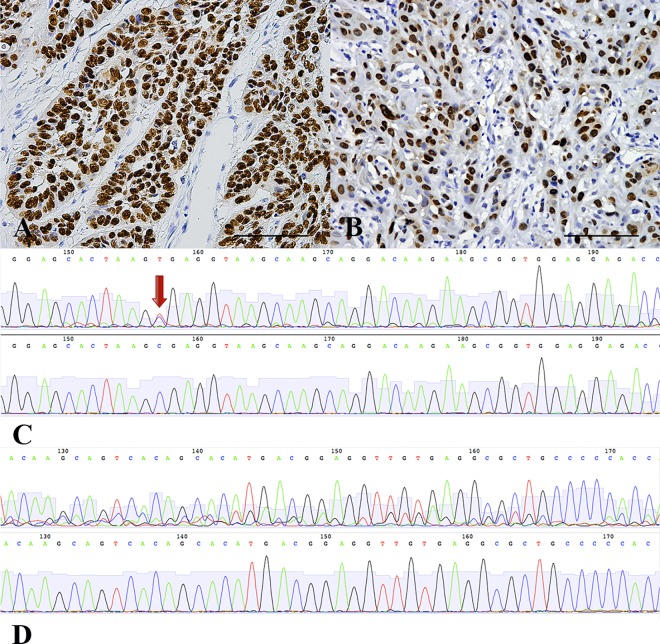
p53 IHC and *TP53* exons 5–8 Sequencing. (A, B) Accumulation of abnormal p53 protein in tumor cell nuclei. (C, D) An atlas of direct sequencing of *TP53* exons 5–8. Upper photographs show different classifications of mutants, and lower photographs are normal controls; *TP53* exons 5–8 point mutation are marked with red arrow in picture C; Heterozygous *TP53* exons 5–8 frame-shift mutations are shown in picture D.

Interestingly, all 22 HPV16-positive samples were p53 negative. To avoid interference from HPV16 E6-mediated p53 degradation, the 22 HPV16-positive OPSCC samples were excluded during the analysis of p53 expression. The results showed that 30.12% (50/166) of the HPV-negative OPSCC cases exhibited positive p53 expression (Fig 3A and 3B). The III-IV clinical stage group had a higher p53-positive rate than the I-II clinical stage group (*p =* 0.036). p53 expression did not have any clear associations with other clinicopathological characteristics, including smoking and drinking (S3 Table).

Next, we selected 43 FFPE samples that had abundant tumor cells and normal control tissues from the 188 OPSSC cases to perform sequencing of *TP53* exons 5–8. Detailed information on the 43 cases is listed in S4 Table. These samples included 27 HPV16-negative cases and 16 HPV16-positive cases. In total, 32.56% (14/43) OPSCC cases had *TP53* gene mutations, and these 14 cases were all HPV negative. Among these 14 cases, 64.29% (9/14) had point mutations (Fig 3C), and the other 5 (35.71%; 5/14) cases had deletion/insertion frame-shift mutations (Fig 3D). Compared with the sequencing results from the control group, all 14 patients had heterozygous mutations in these exons of the *TP53* gene (Fig 3C and 3D). Statistical analysis of the data did not show a significant association between mutations in *TP53* exons 5–8 and smoking, drinking, lymph node metastasis, histological grade, or clinical stage in the 43 OPSCC cases. However, these mutations were closely associated with HPV infection. The p53-positive samples showed a higher mutation rate in *TP53* exons 5–8 (77.78%; 7/9) compared with the p53-negative samples (20.59%; 7/34) (*p =* 0.003) (S5 Table).

### Survival Analysis

**Fig 4:**
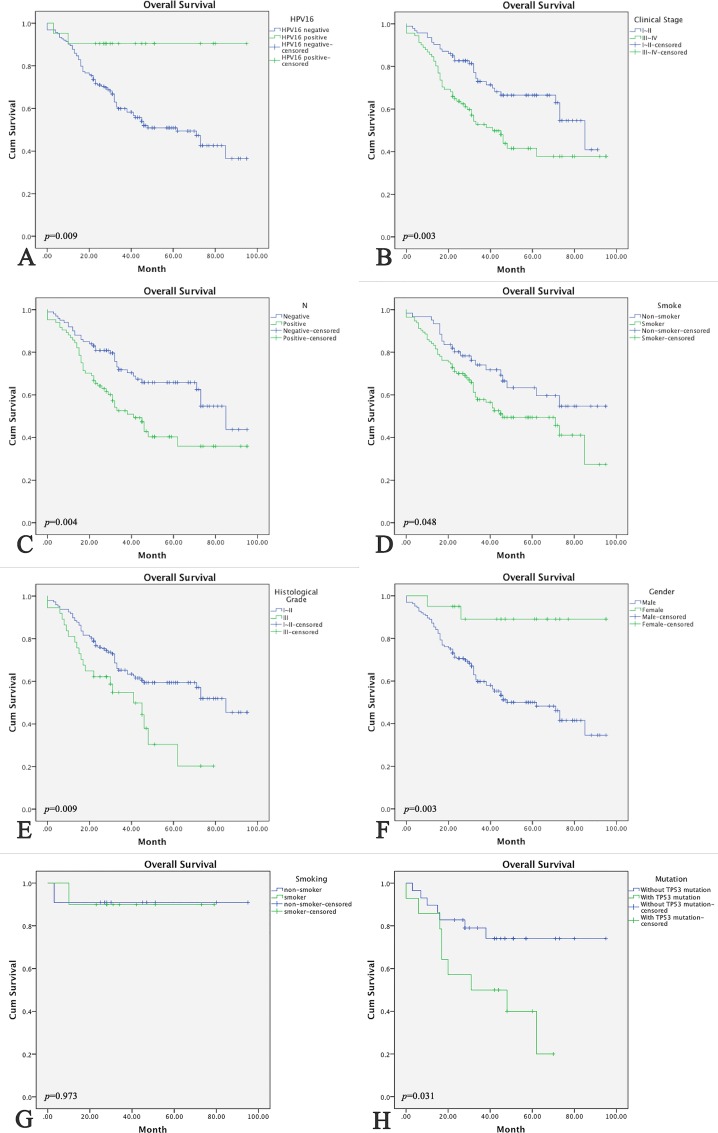
Kaplan–Meier Overall Survival and Survival Curves. (A) HPV status, (B) clinical stage, (C) nodal stage, (D) smoking status, (E) histological grade, and (F) gender were compared using categorical data analysis, and overall-survival was estimated using the Kaplan-Meier method among all 188 OPSCC cases. (G) Smoking status (among 22 HPV-associated OPSCC patients) and (H) *TP53* exons 5–8 mutation status (among 43 sequencing specimen) were compared by categorical data analysis, and overall-survival was estimated using the Kaplan-Meier method.

Follow-up information was obtained for 97.87% (184/188) of the cases. The follow-up period lasted from March 1, 2008, until February 29, 2016. The follow-up periods ranged from 4 to 95 months, and the median time was 33 months. At the end of the follow-up period, 42.93% (79/184) of the patients had passed away. The Kaplan-Meier method was used for survival analysis and to plot the survival curve. For the 188 OPSCC patients, the following characteristics were linked with better overall survival: HPV16 positive, female patients, non-smoker, histological grade I, and N0 stage diseases (*p =* 0.009, 0.003, 0.048, 0.009, and 0.004, respectively) (Fig 4A–4F). The multivariate analysis indicated that HPV16 infection status (*p =* 0.011), histological grade (*p =* 0.017), and N stage (*p =* 0.019) were independent prognostic factors for patients with OPSCC. It is worth noting that in the HPV-associated OPSCC group, there were no significant differences in overall survival between smoking and non-smoking patients (Fig 4G). In addition, among the 43 sequenced specimens, patients without mutations in *TP53* exons 5–8 showed better overall survival (*p =* 0.031) (Fig 4H) (Table 3).

**Table 3:** Kaplan-Meier and Cox-Regression Analysis of the 188 Primary OPSCC Cases.

Variable	Kaplan-Meier	Cox-Regression		
	*p* value	*p* value	Hazard Ratio	95% Confidential Interval
**Sex**	0.003	0.152	0.340	0.078 to 1.489
**Age**	0.833			
**Histological grades**	0.009	0.017	1.916	1.124 to 3.966
**T stage**	0.183			
**Chemotherapy**	0.046	0.112	1.539	0.905 to 2.619
**Radiation therapy**	0.996			
**Metastasis**	0.072			
**Clinical stage**	0.003			
**Nodal stage**	0.004	0.019	1.807	1.101 to 2.964
**Smoke**	0.048	0.661	1.126	0.663 to 1.911
**Alcohol consumption**	0.499			
**Site**	0.378			
**p16INK4A expression**	0.417			
**HPV16**	0.009	0.011	0.159	0.038 to 0.622
**p53 expression**	0.073			

## Discussion

This was the first study conducted in eastern China that retrospectively investigated the relationships between HPV infection status, *TP53* mutations, and prognosis in patients with OPSCC. We found that Chinese OPSCC patients were primarily middle-aged to elderly male patients with smoking and drinking habits, with a peak age of 51–60. This is strikingly different from what is known about OPSCC in the USA, where a significant proportion of younger patients (40–50 years) are seen in addition to those in the 50–60 age group [26,27], although it is consistent with the prevalence of OPSCC in Hong Kong, where patients had a mean age of 59.8 years and more than 50% of whom had a history of smoking and/or drinking [23]. The most frequently involved site of OPSCC in this study was the tongue base, whereas tonsil carcinoma only accounted for 5.24% (10/188) of the total patients. This was quite different from other studies in the USA, Japan, and South Korea showing that over 50% of OPSCC cases were tonsil lesions [19,28,29]. The lower HPV infection rate due to different sexual behaviors between the Chinese population and Western counties could explain the lower prevalence of tonsillar cancer in eastern China [3,30]. Although there was an apparent increase in the prevalence of HPV-associated OPSCC during 2008–2013, the number of HPV-positive patients remained at low absolute levels. These results suggested that there could be differences in OPSCC tumorigenesis between Western and Asian countries due to economic development and lifestyle. Current studies based on Chinese OPSCC patients suggest that both HPV-negative and HPV-positive OPSCC patients did not show a significant decrease in smoking habits [22,23]. Consistent with previous studies by Huang et al. [22] (54.55%; 6/11) and Lam et al. [23] (60.47%; 26/43), we found a high smoking rate (45.45%; 10/22) among HPV-associated OPSCC cases, which was strikingly different from results in Western countries [2–5]. Although the smokers in this study exhibited significantly poorer prognoses in general, this variable had little effect on the outcome of HPV-positive patients. For many decades, smoking has been deemed an important tumor-driven pathogenic factor and is closely related to *TP53* gene dysfunctions in the HNSCC [31,32], although controversies surrounding smoking and HPV-associated OPSCC still remain [33,34]. Our results indicate that smoking may be an important but likely not a decisive risk factor in HPV-associated OPSCC, suggesting that the outcome of OPSCC is primarily determined by distinct pathogenic mechanisms rather than affecting factors. This result is at odds with a recent report of tobacco use in HPV-associated lesions, although it still supports the view that smoking is a crucial cofactor during the progression of this disease [33,35]. Another insight from this study is that HPV-associated OPSCC can be viewed as a unique clinical entity that is distinct from HPV-negative tumors and that HPV infection status is an independent prognostic factor for patients with OPSCC, consistent with the conclusions of previous reports [10,36].

This was also the first study that retrospectively investigated *TP53* dysfunction in the context of OPSCC in China. We found that patients without mutations in exons 5–8 of *TP53* had better prognoses than patients who did carry mutations. This result was not surprising and was consistent with most research based on Western HNSCC [37,38]. However, we did notice certain differences, particularly with respect to the correlations between smoking and alcohol consumption with *TP53* mutations [32]. In this study, *TP53* gene mutations were not significantly associated with smoking or drinking histories, suggesting that there are other risk factors that can cause *TP53* mutations. Therefore, the environment of the patient and other unknown factors must also play important roles in the occurrence and development of this disease in China. Furthermore, the 22 HPV-associated OPSCC patients in this study were all p53 negative; one explanation for this finding could be that HPV infection is the dominant pathogenic factor for HPV-associated OPSCC. Therefore, it is possible that all the HPV-associated cases did not carry *TP53* gene mutations, and the tumor cells expressed only a small amount of wild-type p53 protein with a shorter half-life [34]. Another possibility is that the absence of p53 expression was due to the degradation of accumulated mutant p53 by the HPV16 E6 protein via the ubiquitination pathway or just simply caused by disruptive *TP53* mutation in the nucleus [39]. Based on the sequencing results from the 16 cases of HPV-associated OPSCC, all HPV16-positive Chinese OPSCC smokers with negative p53 expression also did not carry *TP53* mutations. Combined with the high expression levels of p16INK4A, as detected by IHC, we hypothesized that the active biological status of HPV in tumors is the key factor responsible for the characteristic changes in host cells and that HPV16-associated OPSCC is less likely to succumb to synchronous and metachronous tumors. It is also worth mentioning that the mutation rate for *TP53* exons 5–8 in the p53-positive group was significantly higher than in the p53-negative group. Considering the known correlations between *TP53* gene mutations and p53 expression, it is possible that p53 is easier to detect by IHC in patients with *TP53* mutations, which could be used for preliminary screening of *TP53* mutations in FFPE tissues.

Although advanced (e.g., ISH and reverse blot hybridization) and reliable (e.g., PCR and *TP53* exon 5–8 sequencing) complementary testing techniques were applied during the experimental procedures, this study still had several limitations. Only degraded fragments of DNA could be obtained from FFPE samples, which limited our ability to directly sequence all *TP53* exons. Although our oral and maxillofacial tumor center in the Department of Oral and Maxillofacial Surgery at Shanghai 9th People's Hospital is one of the largest clinical centers in China, any retrospective study based on a single-center cohort will not be comprehensive enough to draw definitive conclusions. Further additional randomized, multi-center, long-term follow-up studies will be needed to fully address this topic.

In conclusion, although the HPV16 infection rate was relatively low, infection status was still an independent prognostic predictor in patients with OPSCC. In this study, HPV infection was not only the critical factor in the pathogenesis of OPSCC but also a predominant determining factor in prognosis. The other two independent prognostic factors we found were cervical nodal involvement and histological grade. Aside from HPV infection evaluation, early diagnosis and treatment were equally critical for OPSCC prognosis in this Chinese population. Although high tobacco and alcohol consumption rates are also important, we conclude that HPV16 infection and *TP53* dysfunction are distinct pathogenic factors in eastern Chinese OPSCC patients.

## Supporting Information

S1 Table Primer Sequences and PCR Conditions. (DOC)

S2 Table The Distribution of Various Clinicopathological Features and Sites of 188 Primary OPSCC Cases During 2008–2014. (DOCX)

S3 Table p53 Expression Rate in 166 HPV-negative Primary OPSCC Patients with Various Clinicopathological Features. (DOCX)

S4 Table The Distribution of Clinicopathological Characteristics in 43 Primary OPSCC Cases. (DOCX)

S5 Table Clinicopathological Features and *TP53* Mutation Status in 43 Primary OPSCC Patients. (DOCX)

## References

[1] International Agency for Research on Cancer. Human papillomaviruses. In: IARC monographs on the evaluation of carcinogenic risks to humans Lyon: IARC Press; 2007 pp. 1–670.

[2] Kreimer AR, Clifford GM, Boyle P, Franceschi S. Human papillomavirus types in head and neck squamous cell carcinomas worldwide: a systematic review. Cancer Epidemiol Biomarkers Prev. 2005;14: 467–475. 10.1158/1055-9965.EPI-04-0551 15734974

[3] Chaturvedi AK, Engels EA, Pfeiffer RM, Hernandez BY, Xiao W, Kim E, et al Human papillomavirus and rising oropharyngeal cancer incidence in the United States. J Clin Oncol. 2011;29: 4294–4301. 10.1200/JCO.2011.36.4596 21969503 PMC3221528

[4] Gillison ML, Koch WM, Capone RB, Spafford M, Westra WH, Wu L, et al Evidence for a causal association between human papillomavirus and a subset of head and neck cancers. J Natl Cancer Inst. 2000;92: 709–720. 10.1093/jnci/92.9.709 10793107

[5] Klussmann JP, Weissenborn SJ, Wieland U, Dries V, Kolligs J, Jungehuelsing M, et al Prevalence, distribution, and viral load of human papillomavirus 16 DNA in tonsillar carcinomas. Cancer. 2001;92: 2875–2884. 10.1002/1097-0142(20011201)92:11<2875::aid-cncr10130>3.0.co;2-7 11753961

[6] Herrero R, Castellsague X, Pawlita M, Lissowska J, Kee F, Balaram P, et al Human papillomavirus and oral cancer: the International Agency for Research on Cancer multicenter study. J Natl Cancer Inst. 2003;95: 1772–1783. 10.1093/jnci/djg107 14652239

[7] Applebaum KM, Furniss CS, Zeka A, Posner MR, Smith JF, Bryan J, et al Lack of association of alcohol and tobacco with HPV16-associated head and neck cancer. J Natl Cancer Inst. 2007;99: 1801–1810. 10.1093/jnci/djm233 18042931

[8] Schwartz SM, Daling JR, Doody DR, Wipf GC, Carter JJ, Madeleine MM, et al Oral cancer risk in relation to sexual history and evidence of human papillomavirus infection. J Natl Cancer Inst. 1998;90: 1626–1636. 10.1093/jnci/90.21.1626 9811312

[9] D'Souza G, Kreimer AR, Viscidi R, Pawlita M, Fakhry C, Koch WM, et al Case-control study of human papillomavirus and oropharyngeal cancer. N Engl J Med. 2007;356: 1944–1956. 10.1056/NEJMoa065497 17494927

[10] Ang KK, Harris J, Wheeler R, Weber R, Rosenthal DI, Nguyen-Tan PF, et al Human papillomavirus and survival of patients with oropharyngeal cancer. N Engl J Med. 2010;363: 24–35. 10.1056/NEJMoa0912217 20530316 PMC2943767

[11] Rischin D, Young RJ, Fisher R, Fox SB, Le QT, Peters LJ, et al Prognostic significance of p16INK4A and human papillomavirus in patients with oropharyngeal cancer treated on TROG 02.02 phase III trial. J Clin Oncol. 2010;28: 4142–4148. 10.1200/JCO.2010.29.2904 20697079 PMC2953971

[12] Cabelguenne A, Blons H, de Waziers I, Carnot F, Houllier AM, Soussi T, et al p53 alterations predict tumor response to neoadjuvant chemotherapy in head and neck squamous cell carcinoma: a prospective series. J Clin Oncol. 2000;18: 1465–1473. 10735894 10.1200/JCO.2000.18.7.1465

[13] Snijders PJ, Steenbergen RD, Top B, Scott SD, Meijer CJ, Walboomers JM. Analysis of p53 status in tonsillar carcinomas associated with human papillomavirus. J Gen Virol. 1994;75: 2769–2775. 10.1099/0022-1317-75-10-2769 7931165

[14] Huang H, Li CY, Little JB. Abrogation of P53 function by transfection of HPV16 E6 gene does not enhance resistance of human tumour cells to ionizing radiation. Int J Radiat Biol. 1996;70: 151–160. 10.1080/095530096145148 8794844

[15] Ferris RL, Martinez I, Sirianni N, Wang J, Lopez-Albaitero A, Gollin SM, et al Human papillomavirus-16 associated squamous cell carcinoma of the head and neck (SCCHN): a natural disease model provides insights into viral carcinogenesis. Eur J Cancer. 2005;41: 807–815. 10.1016/j.ejca.2004.11.023 15763658

[16] Chaturvedi AK, Anderson WF, Lortet-Tieulent J, Curado MP, Ferlay J, Franceschi S, et al Worldwide trends in incidence rates for oral cavity and oropharyngeal cancers. J Clin Oncol. 2013;31: 4550–4559. 10.1200/JCO.2013.50.3870 24248688 PMC3865341

[17] Hwang TZ, Hsiao JR, Tsai CR, Chang JS. Incidence trends of human papillomavirus-related head and neck cancer in Taiwan, 1995–2009. Int J Cancer. 2015;137: 395–408. 10.1002/ijc.29330 25395239

[18] Nomura F, Sugimoto T, Kitagaki K, Ito T, Kawachi H, Eishi Y, et al Clinical characteristics of Japanese oropharyngeal squamous cell carcinoma positive for human papillomavirus infection. Acta Otolaryngol. 2014;134: 1265–1274. 10.3109/00016489.2014.944272 25399886

[19] Kawakami H, Okamoto I, Terao K, Sakai K, Suzuki M, Ueda S, et al Human papillomavirus DNA and p16 expression in Japanese patients with oropharyngeal squamous cell carcinoma. Cancer Med. 2013;2: 933–941. 10.1002/cam4.151 24403267 PMC3892398

[20] Mizumachi T, Kano S, Sakashita T, Hatakeyama H, Suzuki S, Homma A, et al Improved survival of Japanese patients with human papillomavirus-positive oropharyngeal squamous cell carcinoma. Int J Clin Oncol. 2013;18: 824–828. 10.1007/s10147-012-0469-6 22936564

[21] Joo YH, Yoo Ie R, Cho KJ, Park JO, Nam IC, Kim MS. Preoperative 18F-FDG PET/CT and high-risk HPV in patients with oropharyngeal squamous cell carcinoma. Head Neck. 2014;36: 323–327. 10.1002/hed.23296 23729374

[22] Huang H, Zhang B, Chen W, Zhou SM, Zhang YX, Gao L, et al Human papillomavirus infection and prognostic predictors in patients with oropharyngeal squamous cell carcinoma. Asian Pac J Cancer Prev. 2012;13: 891–896. 10.7314/APJCP.2012.13.3.891 22631667

[23] Lam EW, Chan JY, Chan AB, Ng CS, Lo ST, Lam VS, et al Prevalence, clinicopathological characteristics, and outcome of human papillomavirus-associated oropharyngeal cancer in southern Chinese patients. Cancer Epidemiol Biomarkers Prev. 2016;25: 165–173. 10.1158/1055-9965.EPI-15-0869 26604268

[24] Singhi AD, Westra WH. Comparison of human papillomavirus in situ hybridization and p16 immunohistochemistry in the detection of human papillomavirus-associated head and neck cancer based on a prospective clinical experience. Cancer. 2010;116: 2166–2173. 10.1002/cncr.25033 20186832

[25] Hafkamp HC, Speel EJ, Haesevoets A, Bot FJ, Dinjens WN, Ramaekers FC, et al A subset of head and neck squamous cell carcinomas exhibits integration of HPV 16/18 DNA and overexpression of p16INK4A and p53 in the absence of mutations in p53 exons 5–8. Int J Cancer. 2003;107: 394–400. 10.1002/ijc.11389 14506739

[26] Chaturvedi AK, Engels EA, Anderson WF, Gillison ML. Incidence trends for human papillomavirus-related and -unrelated oral squamous cell carcinomas in the United States. J Clin Oncol. 2008;26: 612–619. 10.1200/JCO.2007.14.1713 18235120

[27] Shiboski CH, Schmidt BL, Jordan RC. Tongue and tonsil carcinoma: increasing trends in the U.S. population ages 20–44 years. Cancer. 2005;103: 1843–1849. 10.1002/cncr.20998 15772957

[28] Schache AG, Liloglou T, Risk JM, Filia A, Jones TM, Sheard J, et al Evaluation of human papilloma virus diagnostic testing in oropharyngeal squamous cell carcinoma: sensitivity, specificity, and prognostic discrimination. Clin Cancer Res. 2011;17: 6262–6271. 10.1158/1078-0432.CCR-11-0388 21969383 PMC3188400

[29] Sun JR, Kim SM, Seo MH, Kim MJ, Lee JH, Myoung H. Oral cancer incidence based on annual cancer statistics in Korea. J Korean Assoc Oral Maxillofac Surg. 2012;38: 20–28. 10.5125/jkaoms.2012.38.1.20

[30] Hammarstedt L, Lindquist D, Dahlstrand H, Romanitan M, Dahlgren LO, Joneberg J, et al Human papillomavirus as a risk factor for the increase in incidence of tonsillar cancer. Int J Cancer. 2006;119: 2620–2623. 10.1002/ijc.22177 16991119

[31] Cruz I, Snijders PJ, Van Houten V, Vosjan M, Van der Waal I, Meijer CJ. Specific p53 immunostaining patterns are associated with smoking habits in patients with oral squamous cell carcinomas. J Clin Pathol. 2002;55: 834–840. 10.1136/jcp.55.11.834 12401821 PMC1769794

[32] Urashima M, Hama T, Suda T, Suzuki Y, Ikegami M, Sakanashi C, et al Distinct effects of alcohol consumption and smoking on genetic alterations in head and neck carcinoma. PLoS One. 2013;8: e80828 10.1371/journal.pone.0080828 24278325 PMC3835411

[33] Maxwell JH, Kumar B, Feng FY, Worden FP, Lee JS, Eisbruch A, et al Tobacco use in human papillomavirus-positive advanced oropharynx cancer patients related to increased risk of distant metastases and tumor recurrence. Clin Cancer Res. 2010;16: 1226–1235. 10.1158/1078-0432.CCR-09-2350 20145161 PMC2822887

[34] Westra WH, Taube JM, Poeta ML, Begum S, Sidransky D, Koch WM. Inverse relationship between human papillomavirus-16 infection and disruptive p53 gene mutations in squamous cell carcinoma of the head and neck. Clin Cancer Res. 2008;14: 366–369. 10.1158/1078-0432.CCR-07-1402 18223210

[35] Castellsague X, Munoz N. Chapter 3: cofactors in human papillomavirus carcinogenesis—role of parity, oral contraceptives, and tobacco smoking. J Natl Cancer Inst Monogr. 2003;31: 20–28. 10.1093/oxfordjournals.jncimonographs.a003477 12807941

[36] Nichols AC, Faquin WC, Westra WH, Mroz EA, Begum S, Clark JR, et al HPV-16 infection predicts treatment outcome in oropharyngeal squamous cell carcinoma. Otolaryngol Head Neck Surg. 2009;140: 228–234. 10.1016/j.otohns.2008.11.025 19201294

[37] Heaton CM, Durr ML, Tetsu O, van Zante A, Wang SJ. TP53 and CDKN2a mutations in never-smoker oral tongue squamous cell carcinoma. Laryngoscope. 2014;124: E267–273. 10.1002/lary.24595 24431303

[38] Neskey DM, Osman AA, Ow TJ, Katsonis P, McDonald T, Hicks SC, et al Evolutionary action score of TP53 identifies high-risk mutations associated with decreased survival and increased distant metastases in head and neck cancer. Cancer Res. 2015;75: 1527–1536. 10.1158/0008-5472.CAN-14-2735 25634208 PMC4383697

[39] Scheffner M, Huibregtse JM, Vierstra RD, Howley PM. The HPV-16 E6 and E6-AP complex functions as a ubiquitin-protein ligase in the ubiquitination of p53. Cell. 1993;75: 495–505. 10.1016/0092-8674(93)90384-3 8221889

