# Supplementary material for: Human Papillomavirus 16 Infection and TP53 Mutation: Two Distinct Pathogeneses for Oropharyngeal Squamous Cell Carcinoma in an Eastern Chinese Population
Source: PLoS One. 2016 Oct 17;11(10):e0164491. doi: 10.1371/journal.pone.0164491 (PMC5066983; doi:10.1371/journal.pone.0164491)
Supplement: S1 Table — (DOC) [file pone.0164491.s001.doc]

**S1 Table. Primer sequences and** PCR conditions

| Primer | Forward | | Reverse | | Size | |
| --- | --- | --- | --- | --- | --- | --- |
| HPV16 E6 | CTGCGACGTGAGGTATATGACTTT | | ACATACAGCATATGGATTCCCATCT | | 118 bp | |
| HPV18 E6 | AAACCGTTGAATCCAGCAGAA | | GTCGTTCCTGTCGTGCTCG | | 121 bp | |
| β-globin | CAGGTACGGCTGTCATCACTTAGA | | CATGGTGTCTGTTTGACGTTGCTA | | 184 bp | |
| *TP53* Exon5 | CTTGTGCCCTGACTTTCAACTCTGTCTC | | TGGGCAACCAGCCCTGTCGTCTCTCCA | | 270 bp | |
| *TP53* Exon6 | CCAGGCCTCTGATTCCTCACTGATTGCTC | | GCCACTGACAACCACCCTTAACCCCTC | | 202 bp | |
| *TP53* Exon7 | GCCTCATCTTGGGCCTGTGTTATCTCC | | GGCCAGTGTGCAGGGTGGCAAGTGGCTC | | 126 bp | |
| *TP53* Exon8 | GTAGGACCTGATTTCCTTACTGCCTCTTGC | | ATAACTGCACCCTTGGTCTCCTCCACCGC | | 239 bp | |
|  | | PCR conditions | | | |  |
|  | Melting time | | | Anneal/Extension time | | |
| HPV16 E6 | 98°C, 120 s | | | 63°C, 30 s/72°C, 60 s | | |
| HPV18 E6 | 98°C, 120 s | | | 63°C, 30 s/72°C, 60 s | | |
| β-globin | 98°C, 120 s | | | 63°C, 30 s/72°C, 60 s | | |
