# Supplementary material for: Human Papillomavirus 16 Infection and TP53 Mutation: Two Distinct Pathogeneses for Oropharyngeal Squamous Cell Carcinoma in an Eastern Chinese Population
Source: PLoS One. 2016 Oct 17;11(10):e0164491. doi: 10.1371/journal.pone.0164491 (PMC5066983; doi:10.1371/journal.pone.0164491)
Supplement: S2 Table — (DOCX) [file pone.0164491.s002.docx]

**S2 Table. The distribution of various clinicopathological features and sites of 188 primary OPSCC cases during 2008–2014**

| **Patient/tumor data** | **Tumor site, n** | | | | **Total, n(%)** |
| --- | --- | --- | --- | --- | --- |
|  | **Base of tongue** | **Soft palate** | **Tonsil** | **Oropharynx (not further specified)** |  |
| **Age at diagnosis, y** |  |  |  |  |  |
| <60 | 41 | 29 | 9 | 34 | 113(59.20) |
| ≥60 | 34 | 21 | 1 | 22 | 78(40.80) |
| **Sex** |  |  |  |  |  |
| Male | 66 | 47 | 7 | 51 | 171(89.50) |
| Female | 9 | 3 | 3 | 5 | 20(10.50) |
| **Smoking** |  |  |  |  |  |
| Smoker | 39 | 34 | 4 | 42 | 119(62.30) |
| Nonsmoker | 28 | 15 | 6 | 13 | 62(32.50) |
| Unknown | 8 | 1 | 0 | 1 | 10(5.20) |
| **Alcohol consumption** |  |  |  |  |  |
| Drinker | 31 | 23 | 3 | 33 | 90(47.10) |
| Nondrinker | 36 | 26 | 7 | 22 | 91(47.70) |
| Unknown | 8 | 1 | 0 | 1 | 10(5.20) |
| **Pathological grades** |  |  |  |  |  |
| 1 | 5 | 10 | 0 | 5 | 20(10.50) |
| 2 | 56 | 31 | 6 | 40 | 133(69.60) |
| 3 | 14 | 9 | 4 | 11 | 38(19.90) |
| **Nodal stage** |  |  |  |  |  |
| Negative | 39 | 28 | 3 | 34 | 104(54.50) |
| Positive | 36 | 22 | 7 | 22 | 87(45.50) |
| **Clinical stage** |  |  |  |  |  |
| I~II | 36 | 27 | 3 | 31 | 97(50.80) |
| II~IV | 39 | 23 | 7 | 25 | 94(49.20) |
| **HPV status** |  |  |  |  |  |
| HPV Negative | 69 | 47 | 3 | 50 | 169(88.50) |
| HPV Positive | 6 | 3 | 7 | 6 | 22(11.50) |
| **Total, n (%)** | 75(39.3) | 50(26.2) | 10(5.2) | 56(29.3) | 191(100) |
