# Supplementary material for: Human Papillomavirus 16 Infection and TP53 Mutation: Two Distinct Pathogeneses for Oropharyngeal Squamous Cell Carcinoma in an Eastern Chinese Population
Source: PLoS One. 2016 Oct 17;11(10):e0164491. doi: 10.1371/journal.pone.0164491 (PMC5066983; doi:10.1371/journal.pone.0164491)
Supplement: S3 Table — (DOCX) [file pone.0164491.s003.docx]

**S3 Table. p53 expression rate in 166 HPV16/18-negative primary OPSCC patients with various clinicopathological features**

|  | **p53 positive** | | **p53 negative** | **Total** | **Statistical significance** |
| --- | --- | --- | --- | --- | --- |
| **Patient/tumor data** |  |  | |  |  |
| No. of patients | 50 | 116 | | 166 |  |
| Unknown |  |  | | 3 |  |
| **Age at diagnosis, y** |  |  | |  |  |
| Mean | 59.68 | 58.49 | | 58.85 | NS |
| Median | 59.5 | 57.5 | | 58 |  |
| **Sex** |  |  | |  |  |
| Male | 47 | 105 | | 152 | NS |
| Female | 3 | 11 | | 14 |  |
| Unknown |  |  | | 3 |  |
| **Tumor site** |  |  | |  |  |
| Base of tongue | 21 | 47 | | 68 | NS |
| Oropharynx (not further specified) | 14 | 34 | | 48 |  |
| Soft palate | 14 | 33 | | 47 |  |
| Tonsil | 1 | 2 | | 3 |  |
| Unknown |  |  | | 3 |  |
| **Smoking** |  |  | |  |  |
| Smoker | 37 | 68 | | 105 | NS |
| Nonsmoker | 13 | 39 | | 52 |  |
| Unknown |  |  | | 12 |  |
| **Alcohol consumption** |  |  | |  |  |
| Drinker | 29 | 54 | | 83 | NS |
| Nondrinker | 21 | 53 | | 74 |  |
| Unknown |  |  | | 12 |  |
| **Pathological grades** |  |  | |  |  |
| 1 | 2 | 17 | | 19 | NS |
| 2 | 37 | 81 | | 118 |  |
| 3 | 11 | 18 | | 29 |  |
| Unknown |  |  | | 3 |  |
| **Nodal stage** |  |  | |  |  |
| Negative | 25 | 70 | | 95 | NS |
| Positive | 25 | 46 | | 71 |  |
| Unknown |  |  | | 3 |  |
| **Clinical stage** |  |  | |  |  |
| I~II | 20 | 67 | | 87 | 0.036 |
| III~IV | 30 | 49 | | 79 |  |
| Unknown |  |  | | 3 |  |

Information concerning smoking and drinking was not available for 10 patients, one of whom was HPV positive.

p53 IHC could not be performed in three cases because the entire specimen had been used in previous experiments.

NS, Not Significant
