# Supplementary material for: Human Papillomavirus 16 Infection and TP53 Mutation: Two Distinct Pathogeneses for Oropharyngeal Squamous Cell Carcinoma in an Eastern Chinese Population
Source: PLoS One. 2016 Oct 17;11(10):e0164491. doi: 10.1371/journal.pone.0164491 (PMC5066983; doi:10.1371/journal.pone.0164491)
Supplement: S4 Table — (DOCX) [file pone.0164491.s004.docx]

**S4 Table. The distributions of clinicopathological characteristics in 43 primary OPSCC cases**

| **Patient/tumor data** | **Sex** | **Age** | **Pathological grades** | **TNM stage** | **Smoker/Alcohol consumption** | **Tumor site** | ***TP53* Mutation** | **p53 positive cell percentage** |
| --- | --- | --- | --- | --- | --- | --- | --- | --- |
| Ne1 | M | 42 | II~III | T1N2aM0 | S&Y | SP | N | 60% |
| Ne2 | M | 41 | I~II | T3N2cM0 | S&Y | OR | P | 0% |
| Ne3 | F | 80 | I~II | T2N0M0 | N | BT | N | 0% |
| Ne4 | M | 53 | I~II | T2N2cM0 | D | BT | FS | 0% |
| Ne5 | M | 58 | II | T2N1M0 | S&Y | SP | FS | 10% |
| Ne6 | M | 52 | II | T2N2aM0 | S&Y | OR | P | 90% |
| Ne7 | M | 48 | I | T2N0M0 | N | SP | N | 0% |
| Ne8 | M | 64 | I~II | T3N2cM0 | S | BT | P | 40% |
| Ne9 | M | 63 | II~III | T2N1M0 | S | OR | N | 0% |
| Ne10 | M | 51 | II | T2N0M0 | S&Y | OR | FS | 0% |
| Ne11 | M | 47 | I~II | T2N1M0 | D | BT | N | 0% |
| Ne12 | M | 55 | I~II | T2N0M0 | S&Y | OR | N | 0% |
| Ne13 | M | 58 | I~II | T1N2bM0 | S&Y | BT | N | 0% |
| Ne14 | M | 48 | I~II | T1N0M0 | Unknown | OR | N | 5% |
| Ne15 | M | 57 | I | T2N0M0 | S&Y | SP | N | 0% |
| Ne16 | M | 76 | II | T2N2bM0 | N | SP | FS | 0% |
| Ne17 | M | 59 | II | T2N0M0 | S&Y | TO | P | 60% |
| Ne18 | M | 70 | II~III | T1N2M0 | S&Y | SP | N | 75% |
| Ne19 | M | 43 | II~III | T2N1M0 | N | BT | P | 95% |
| Ne20 | M | 56 | II | T2N1M0 | N | BT | P | 65% |
| Ne21 | M | 59 | II | T1N1M0 | N | BT | N | 0% |
| Ne22 | M | 57 | II~III | T4N2bM0 | S | SP | N | 0% |
| Ne23 | M | 66 | I~II | T4N0M0 | S&Y | OR | P | 85% |
| Ne24 | M | 50 | I~II | T2N0M0 | S&Y | OR | N | 0% |
| Ne25 | M | 69 | II | T1N0M0 | S | OR | P | 5% |
| Ne26 | M | 53 | III | T1N2aM0 | N | OR | FS | 0% |
| Ne27 | M | 48 | II | T2N2aM0 | S | OR | P | 60% |
| P1 | F | 51 | II | T2N2aM0 | N | SP | N | 0% |
| P2 | M | 45 | I~II | T2N1M0 | N | TO | N | 0% |
| P3 | M | 64 | III | T2N2aM0 | S | SP | N | 0% |
| P4 | F | 59 | III | T3N1M0 | N | BT | N | 0% |
| P5 | F | 54 | II | T2N2cM0 | N | OR | N | 0% |
| P6 | F | 47 | II | T2N1M0 | N | TO | N | 0% |
| P7 | M | 64 | II | T1N0M0 | S&Y | OR | N | 0% |
| P8 | M | 66 | II | T2N3M0 | S&Y | BT | N | 0% |
| P9 | M | 71 | II | T2N0M0 | N | BT | N | 0% |
| P10 | M | 61 | II~III | T1N0M0 | S&Y | OR | N | 0% |
| P11 | M | 50 | III | T2N0M0 | N | TO | N | 0% |
| P12 | M | 59 | II~III | T1N0M0 | S | SP | N | 0% |
| P13 | M | 57 | III | T2N0M0 | S&Y | OR | N | 0% |
| P14 | F | 58 | III | T2N1M0 | N | TO | N | 0% |
| P15 | M | 60 | I~II | T2N3M0 | N | BT | N | 0% |
| P16 | M | 49 | II | T1N2aM0 | S&Y | OR | FS | 0% |

| Abbreviations: Ne, HPV16/18 negative; P, HPV16 positive; M, male; F, female; S, smoker; D, Drinker; SP, Soft Palate; OR, Oropharynx; BT, Base of Tongue; To, Tonsil; N, None; Point mutation cases are marked blue, whereas deletion/insertion frame-shift mutation cases are marked red. |
| --- |
