# Supplementary material for: Human Papillomavirus 16 Infection and TP53 Mutation: Two Distinct Pathogeneses for Oropharyngeal Squamous Cell Carcinoma in an Eastern Chinese Population
Source: PLoS One. 2016 Oct 17;11(10):e0164491. doi: 10.1371/journal.pone.0164491 (PMC5066983; doi:10.1371/journal.pone.0164491)
Supplement: S5 Table — (DOCX) [file pone.0164491.s005.docx]

**S5 Table. Clinicopathological features and *TP53* mutation status in 43 primary OPSCC patients**

| **Patient/tumor data** | ***TP53* Mutation** | **Non*-TP53* Mutation** | **Overall** | **Statistical significance** |
| --- | --- | --- | --- | --- |
| **Age at diagnosis, y** |  |  |  |  |
| ≥60 | 4 | 9 | 13 | NS |
| <60 | 10 | 20 | 30 |  |
| **Sex** |  |  |  |  |
| Male | 14 | 23 | 37 | NS |
| Female | 0 | 6 | 6 |  |
| **Tumor site** |  |  |  |  |
| Base of tongue | 4 | 8 | 12 | NS |
| Oropharynx (not further specified) | 7 | 9 | 16 |  |
| Soft palate | 2 | 8 | 10 |  |
| Tonsil | 1 | 4 | 5 |  |
| **Smoking** |  |  |  |  |
| Smoker | 9 | 15 | 24 | NS |
| Nonsmoker | 5 | 13 | 18 |  |
| Unknown | 0 | 0 | 1 |  |
| **Alcohol consumption** |  |  |  |  |
| Drinker | 7 | 12 | 19 | NS |
| Nondrinker | 7 | 16 | 23 |  |
| Unknown | 0 | 0 | 1 |  |
| **Pathological grades** |  |  |  |  |
| 1 | 0 | 2 | 2 | NS |
| 2 | 12 | 16 | 28 |  |
| 3 | 2 | 11 | 13 |  |
| **Nodal stage** |  |  |  |  |
| Positive | 10 | 17 | 27 | NS |
| Negative | 4 | 12 | 16 |  |
| **Clinical stage** |  |  |  |  |
| I~II | 2 | 13 | 15 | NS |
| III~IV | 12 | 16 | 28 |  |
| **p53 IHC** |  |  |  |  |
| p53 Positive | 7 | 2 | 9 | *p=*0.003 |
| p53 Negative | 7 | 27 | 34 |  |

NS, Not Significant
